# Understanding Animal Abuse and How to Intervene with Children and Young People: A Practical Guide for Professionals Working with People and Animals

**DOI:** 10.1017/awf.2024.14

**Published:** 2024-03-12

**Authors:** Karianne Muri

**Affiliations:** Senior scientist in animal welfare, Department of Animal Health and Food Safety, Norwegian Veterinary Institute, Ås, Norway

I felt honoured when asked to review this book, because it deals with such immensely important topics, which I spend a considerable proportion of my time talking about to vets and professionals from a range of other disciplines. The stated aim of this book is to be a practical toolkit for professionals working with people and/or animals, to help them *understand, prevent,* and *intervene* in cases of animal abuse.

This book has ten chapters that can be read either in succession or as stand-alone texts. Each chapter ends with questions to consider, conclusions, and key messages. The first five chapters present background information on animal abuse (Chapter 1), psychological risk factors for animal abuse (Chapter 2), the links between animal abuse and domestic violence (Chapter 3), definitions of animal cruelty (Chapter 4), and finally investigation and intelligence sharing (Chapter 5). The next two chapters summarise what can be done about animal abuse, depending on your role: as either a veterinary surgeon (Chapter 6), or a parent or professional working with children (Chapter 7). Chapter 8 describes approaches to deal with both intentional and unintentional animal abuse. Chapter 9 describes how animal welfare interventions can (and should) be evaluated, while the final chapter summarises what we know, what we can do, as well as the knowledge gaps.

The first chapter, *Animal abuse: A concern for all,* is written by the editors, Gilly Mendes Ferreira from the Scottish SPCA, and Joanne M Williams, professor of applied developmental psychology at the University of Edinburgh. This chapter defines animal abuse and outlines *who* has a role in animal abuse cases. The answer is, as the title suggests; this book is for *all who might come across animal abuse* in their work. Ferreira and Williams review research that has highlighted an important concern; that child protection workers rarely ask questions about companion animals in the families they encounter. Key terms are defined and the Scottish SPCA’s ANIMAL approach, which can be used in animal abuse incidents is described. The Animal WISE Footprint Framework (promoting positive human-animal relationships) is also introduced (https://www.scottishspca.org/sites/default/files/2021-10/AnimalWISE%20roadmap%20Oct%202021.pdf) but unfortunately, the Animal WISE figure in the paperback version has a font size and colour that is almost illegible. The authors describe how animals may come to harm due to complex situations for owners, and the roles of each of the relevant professions are explained.

Chapter 2, *Psychological risk factors for animal harm and abuse among children and young people*, is written by Joanne M Williams and Laura M Wauthier (Clinical Psychology, University of Edinburgh). They describe the different roles that companion animals play in children’s lives and discuss how fine the balance can be between positive interactions and inadvertently negative interactions with the potential for harm. The authors then go on to explain how childhood animal abuse can be part of a spectrum of possible interactions, nested in social contexts. Animal abuse conducted by children can be associated with a wide range of factors, and we are presented with current research on the matter. This gives a thorough background for understanding the phenomenon, and the authors encourage a compassionate approach to both the children and the animals.

Chapter 3, *The links between animal abuse and domestic violence/abuse*, is written by Phil Arkow, the co-ordinator of the National Link Coalition and editor of a monthly LINK-Letter. His amazing work within this field will be well-known by many. He describes succinctly how the emotional attachment of abuse victims to their companion animals make the animals “soft targets” for manipulation and can be used to coerce and control family members. He presents the scope of domestic abuse in the US (and the numbers are staggering!). In this chapter, readers will also learn how threats against animals’ safety may be a barrier against leaving a dangerous home or may make abuse victims return to the abuser. The shortage of refuges/shelters that accept companion animals is therefore a threat not only to animals’ safety but also to the safety of the human family members. The role of a companion animal as emotional support for abuse victims is sadly also a vulnerability that abusers can exploit, as Arkow explains. The dynamics of how companion animals become part of different aspects of coercion and abuse is illustrated with The National Link Coalition’s Power and Control Wheel and a review of the related literature. Arkow also describes how devastating domestic violence and witnessing animal abuse is for children. At the end of the chapter there is a long list of implications for each of the professional sectors that may be involved.

In the fourth chapter, Angus Nurse, head of the Department of Criminology and Criminal Justice at Nottingham Trent University, describes how animal cruelty is defined within an international framework. Notions of what constitutes animal abuse are socially constructed, and thus, inevitably vary, as he points out. Nevertheless, in most jurisdictions, animal abuse is now a legal offence. Legal definitions of animal abuse often have deliberate, physical harm as the core element, but readers are reminded that unintentional harm also has the potential to cause severe suffering. The term “unnecessary suffering” is discussed, and both physical harm and psychological stress may fall within the definition of this. The author maintains that although animal welfare legislation may be strong, its enforcement is often more challenging.

Chapter 5, written by Mike Flynn (Chief Superintendent of the Scottish SPCA) and editor Gilly Mendes Ferreira, covers the investigative stage of animal abuse cases. They focus on the work of the SPCSs around the world, with the Scottish SPCA and RSPCA as examples. There are multiple case examples, some of which demonstrate the links between animal abuse and domestic abuse, thus emphasising the need to share intelligence between agencies. Multi-agency tools, such as the Dash Risk Checklist are briefly described.

Chapter 6, which is written by Freda Scott-Park (veterinary surgeon and former Chair of The Links Group UK) moves on to what can be done about animal abuse from a veterinary perspective. The history of how the understanding of the diagnosis of non-accidental injury (NAI) developed, from the pioneering work of Helen Munro and Mike Thrusfield (2001), through collaboration with human health professionals and the founding of The Links Group UK, is provided. Scott-Park explains that veterinary teams all over the UK now have access to a training programme, and many courses are online and therefore available internationally. IVC Evidensia’s profession-wide support network for vets with concerns about abuse is also mentioned (this is a great initiative that I would love to see adopted in other countries as well). The diagnosis of NAI is inherently difficult, and Scott-Park reminds us that the most important step in diagnosing NAI is thinking about it in the first place. She goes through the indicators of animal abuse, then provides advice on how to approach cases. The chapter includes examples of practice protocols for both animal abuse and disclosure of human abuse and presents the A R D R-approach (Ask, Reassure, Document, and Refer). There is also a discussion of the potential dilemmas between codes of professional conduct and *doing the right thing*, which may sometimes involve breaching client confidentiality. The last part of this chapter is about the role vets can play in animal abuse investigations, then it ends with a useful role-play exercise.

The seventh chapter is written by editor Joanne M Williams and covers what parents and professionals working with children can do to prevent childhood animal abuse, and how to intervene, with emphasis on inter-agency efforts. Children who have experienced abuse and/or neglect are very vulnerable, yet views on what is important to these children, Williams explains, rarely includes pets, despite pets often playing a key role in emotional support. I particularly liked the author’s suggestion about how primary school teachers can make animal abuse prevention a natural part of teaching across the existing curriculum. We also learn why social workers should care about animal abuse, and how they can prevent and intervene. Health professionals play a role whenever an animal is implicated in a child’s health condition, and mental health professionals may pick up on worrying behaviours towards animals. However, this presupposes that this subject is specifically asked about, which – unfortunately – often is not the case. Maybe this book will contribute towards a change.

Chapter eight is authored by co-editor, Gilly Mendes Ferreira, and aims to highlight available intervention programmes, particularly for children, also describing the gaps in this area. The chapter also provides recommendations about how to prevent abuse from happening in the first place. The differences between intentional and unintentional animal abuse mean that they need to be dealt with differently – the latter often possible to address through educational programmes, whilst the former requires more targeted intervention. The reader is challenged to consider whether three cases of abuse are correctly categorised as intentional or unintentional (the table’s rightmost column is wrongly headed as “Intervention response”, though). Ferreira explains that animal welfare education should include the exposure of children to positive interactions with animals, rewarding positive behaviours, and correcting negative ones. The four key principles of the Scottish SPCA’s Animal WISE initiative are described (Watch, Inform, Support, and Encourage) with the aim of tackling both intentional and unintentional abuse. The AniCare® model which is offered in parts of the US is also introduced as the only example of a *psychological* intervention programme, and targets both adults and children. For children, the approach includes highly supervised animal assisted therapy, puppet role-play, and emotion-based exercises. The last part of the chapter emphasises the need to evaluate the impacts of intervention programmes. This creates a bridge to the penultimate chapter, which provides a toolkit for the evaluation of interventions – and I would recommend reading these two chapters together. This is written by Janine C Muldoon, University of Edinburgh, together with editor Joanne M Williams. They recommend evaluating an intervention’s reach/engagement and how it works in practice, in addition to the impact in terms of change. The benefits of planning interventions and evaluations together are explained well. Indeed, a logic model of how change is expected to come about is an important part of the development of an intervention. The outcomes of such a model are what you want to measure changes in, in order to assess impact. A crucial problem is finding appropriate and standardised measures, which the authors also discuss briefly. Thus, the chapter provides a useful, albeit not very specific toolkit regarding *how* to measure changes, e.g. in psychological traits, such as attitudes and empathy.

The editors summarise in the final chapter what we know, what we can do, and what we need to know. The importance of inter-agency working is yet again emphasised; we *all* have a role to play. The authors point to increasing challenges with online exposure to animal abuse footage, potentially normalising violence and highlight that few interventions are designed specifically for adolescents. Of new directions for research, they call for more knowledge about psychological risk factors that are amenable to intervention, as well as validated measures that are predictive of behaviour towards animals.

Overall, the authors have based their texts on up-to-date research from this growing field, bridging disciplines such as social sciences and psychology with animal welfare science. This book does not provide an in-depth review of all aspects, which is fine; it is meant to be a *practical guide*, and as that, it certainly succeeds. The readers are provided with sufficient information to find out more elsewhere. One minor drawback, from an international perspective, is that the book focuses much on the UK in terms of relevant agencies, investigations, available intervention programmes etc. Nevertheless, I am certain that readers from other countries and a range of professions will find the book both useful and inspiring – I know I did!

